# Resource use, niche width, and trophic position reveal diverse trophic structure in a tidal freshwater zone fish community

**DOI:** 10.1111/jfb.16057

**Published:** 2025-02-25

**Authors:** Emma E. Bowser, Tyler D. Tunney, Cindy Breau, Brian Hayden

**Affiliations:** ^1^ Canadian Rivers Institute, Department of Biology University of New Brunswick Fredericton New Brunswick Canada; ^2^ Fisheries and Oceans Canada Gulf Fisheries Centre Moncton New Brunswick Canada

**Keywords:** diadromous, food web, isotopic niche, mobile consumer, resource use, stable isotopes, transition zone, trophic position

## Abstract

The tidal freshwater zone is an aquatic transition zone that links a river to its estuary and provides an important habitat used in the life cycle of resident and migratory fishes. Yet, information on the trophic structure of fishes in this habitat is scarce. To address this gap, we characterize the trophic structure of a fish community in the tidal freshwater zone of the Northwest Miramichi River (New Brunswick, Canada). Stable isotope analyses (δ^13^C, δ^15^N, and δ^34^S) of 17 fish species revealed diverse feeding strategies. Resource use varied across species; some fish relied on either marine or freshwater resources, whereas others integrated resources from both habitats. Fishes varied in their trophic position (range 3.1–4.2) which increased with reliance on marine‐derived resources. Species isotopic niche widths varied widely (range 20.4 to 1015.3). Species with intermediate marine resource use (~0.50) had a mix of wide and narrow isotopic niche widths, contrary to wide niches predicted by current literature. Our findings indicate that these narrow‐niched species may either act as a sort of tidal freshwater transition zone specialist or assimilate equal proportions of marine and freshwater resources on average. Trophic information from the tidal freshwater zone improves the understanding of fish communities and food web structure where freshwater rivers and marine ecosystems meet.

## 1 INTRODUCTION

Transition zone habitats are important structural elements of ecosystems that mark a gradual or abrupt change from one ecological unit (e.g., habitat or community) to another (Kark, 2012; P. A. Delcourt & H. R. Delcourt, 1992). Often, these zones are not boundaries, but unique habitats that interact with larger adjacent ecosystems to create key habitat (i.e., spawning, feeding, staging, overwintering areas) for the mobile consumers that exchange resources across the landscape (Elliot et al. 2019; Hanson & Courtenay, 2020; Odum, 1953). These interactions form complex food webs that can bolster ecological function and resilience, underscoring the importance of transition zones in habitat‐based management practices (Polis et al., 1997; Samways et al., 2018; Sánchez‐Hernández & Amundsen, 2018). Understanding the trophic structure of transition zones can improve our understanding of these fundamental ecological processes. Though transition zones are a key habitat for many organisms, often food web structure in these zones is not well studied compared to larger adjacent habitats.

Despite ample research on some transition zones (i.e., estuaries), the tidal freshwater zone is an important aquatic transition zone that has been overlooked in past research. Located between a river's head of tide and the oligohaline region of its estuary, the tidal freshwater zone marks the transition from freshwater to marine habitat, and its tides facilitate the exchange of nutrients and materials between both spaces (Barendregt & Swarth, 2013; Jones et al., 2020; Odum, 1988). Tidal freshwater zones experience a greater depth increase at high tide because their channel width is narrower than that of an estuary, which forces water levels to rise (Odum, 1988). Tidal freshwater zones are associated with large rivers, and they mediate the flow of diadromous fish migrants, freshwater species at the edge of their habitat, and resident species (Odum, 1988; Polis et al., 1997; Samways et al., 2018). Nevertheless, there is limited information on the ecology of tidal freshwater zones, and studies on their trophic structure are particularly scarce. This represents an important knowledge gap as there is considerable interest in the role of fish communities and mobile consumers in the reciprocal exchange of materials and energy between freshwater and marine ecosystems (St George et al., 2024).

Although characterizing trophic interactions among fishes can be highly complex and require considerable resources, key elements of the trophic structure can be rapidly revealed through stable isotope analyses. Natural variation in the ratios of ^13^C/^12^C, ^34^S/^32^S, and ^15^N/^14^N across food web baselines can elucidate whether consumers assimilate nutrients from freshwater, marine, or combined sources (Fry, 2006; Nelson, 2011; Thode, 1991). These differences in δ^13^C, δ^15^N, and δ^34^S enrichment across marine and freshwater habitat enable researchers to estimate the proportion of marine or freshwater resources assimilated by consumers (Phillips et al., 2014). Using three isotopes increases the breadth of ecological information derived from this ecosystem (Connolly et al., 2004; Doubleday et al., 2018). Stable isotope mixing models (SIMM) infer consumer resource use (proportion of basal resources assimilated) by relating the stable isotope ratios of fishes to those of invertebrates (Post, 2002a). Isotopic niche (a coarse proxy for dietary niche) characterizes the range of resources used by describing the region a consumer population occupies in iso‐space (Bearhop et al., 2004; Hette‐Tronquart, 2019; Newsome et al., 2007). Trophic position (a hierarchal measure of the number of trophic transfers from a food web's base to the consumer species) can be estimated using nitrogen isotopes, which exhibit a stepwise enrichment of δ^15^N (approximately 3.4‰ per trophic level with trophic transfer) due to high fractionation (Post, 2002a).

These metrics of trophic structure (e.g., resource use, niche width, and trophic position) can be used not only to provide insights on the dietary habits of a species but also their role in the broader food web (Hooper et al., 2005; Lesser et al., 2020; Vander Zanden et al., 2016). Research suggests that when a species exhibits alterations in one trophic metric (i.e., its resource use), these changes can impact other traits (i.e., niche width). For example, in a study on flood plain ecosystems, Pool et al. (2017) found that fishes that increased their consumption of terrestrial resources during the wet season had wider niches. Similarly, Hayden et al. (2019) examined a large dataset of marine fish populations and found a link between expansion of fish niche width and feeding at an intermediate trophic position. Sánchez‐Hernández and Amundsen (2018) found the fish trophic position is influenced by habitat type, whereby the trophic position of anadromous fishes migrating seaward increased linearly with marine resource use. These metrics of trophic structure are connected both to each other and the ecosystem at large. Studying the relationship between such traits could foreshadow broader impacts on whole ecosystems. However, trophic structure is strongly influenced by habitat; therefore, any relationship between metrics of the trophic structure will likely differ in strength across habitats (Sánchez‐Hernández & Amundsen, 2018). Whether this idea holds true in a transition zone remains to be explored.

**FIGURE 1:**
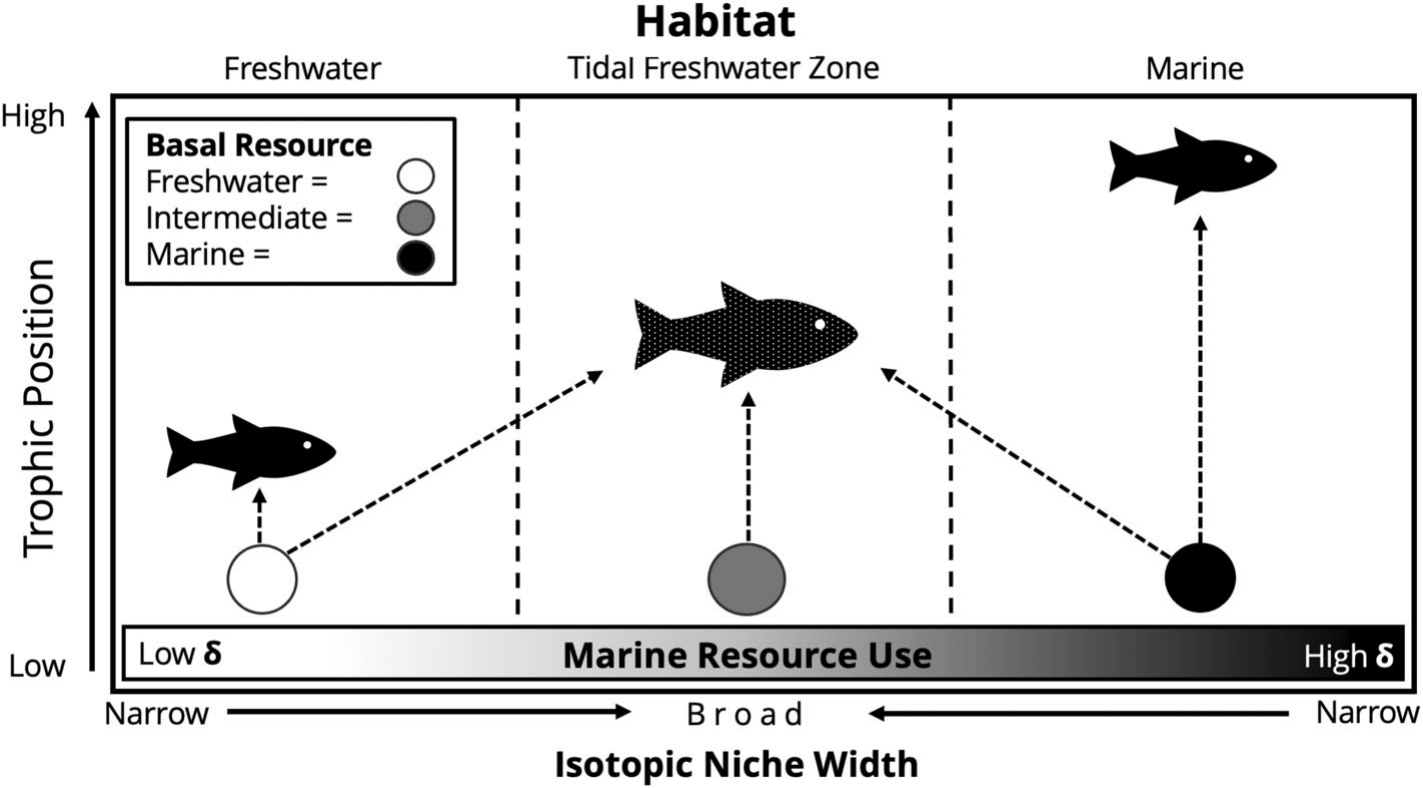
Hypothesized trophic structure for fishes in the tidal freshwater zone. Freshwater, tidal freshwater, and marine habitat is delineated by dotted lines. Basal resource pools of each zone are represented by colored circles. Marine resource use is depicted along the bottom horizontal axis as a gradient of relative stable isotope enrichment. Isotopic niche width is depicted by arrows along bottom axis, fish silhouette size, and dotted texture. Trophic position is depicted on the vertical axis. Species assimilating freshwater resources should exhibit narrow isotopic niches and lower trophic positions; species with intermediate resources use should exhibit broad isotopic niches and intermediate trophic position; species assimilating a high proportion of marine resources should exhibit narrow isotopic niche widths and higher overall trophic position.

Our study documents the trophic structure of a community of fishes in a tidal freshwater transition zone habitat of the Northwest Miramichi River, Canada. We use stable isotopes of carbon (δ^13^C), nitrogen (δ^15^N), and sulfur (δ^34^S) to estimate marine resource use, isotopic niche width, and trophic position of fishes inhabiting the tidal freshwater zone and assess how these trophic traits vary among species. We tested three predictions regarding associations among these trophic traits (Figure 1). We expect (1) a hump‐shaped relationship between resource use and isotopic niche width, where consumer species assimilating a near equal mix from both resource pools should exhibit broader isotopic niches, whereas fishes primarily assimilating from one pool will have narrower niches; (2) a hump‐shaped relationship between isotope niche width and trophic position where consumers with broad isotopic niches should have intermediate trophic positions (i.e., an omnivory effect with increasing niche size); (3) a linear relationship between resource use and trophic position where species with high marine resource use will have higher trophic positions due to longer food chain lengths in marine environments compared to freshwater. Finally, we consider similarities in the trophic role of community members by relating all three metrics using a multivariate analysis.

## 2 METHODS

### 2.1 Ethics statement

Our study followed animal welfare guidelines, laws, and policies outlined in Section 52 of the Fisheries Act. Individual capture and euthanasia of fishes was approved by the Department of Fisheries and Oceans Maritimes and Gulf region (license numbers SG‐RHQ‐19‐502 and SG‐RHQ‐21‐500) and the HMSC Regional Animal Care Committee (Animal Use Protocol numbers 19–34 and 21–35).

### 2.2 Study site and design

**FIGURE 2:**
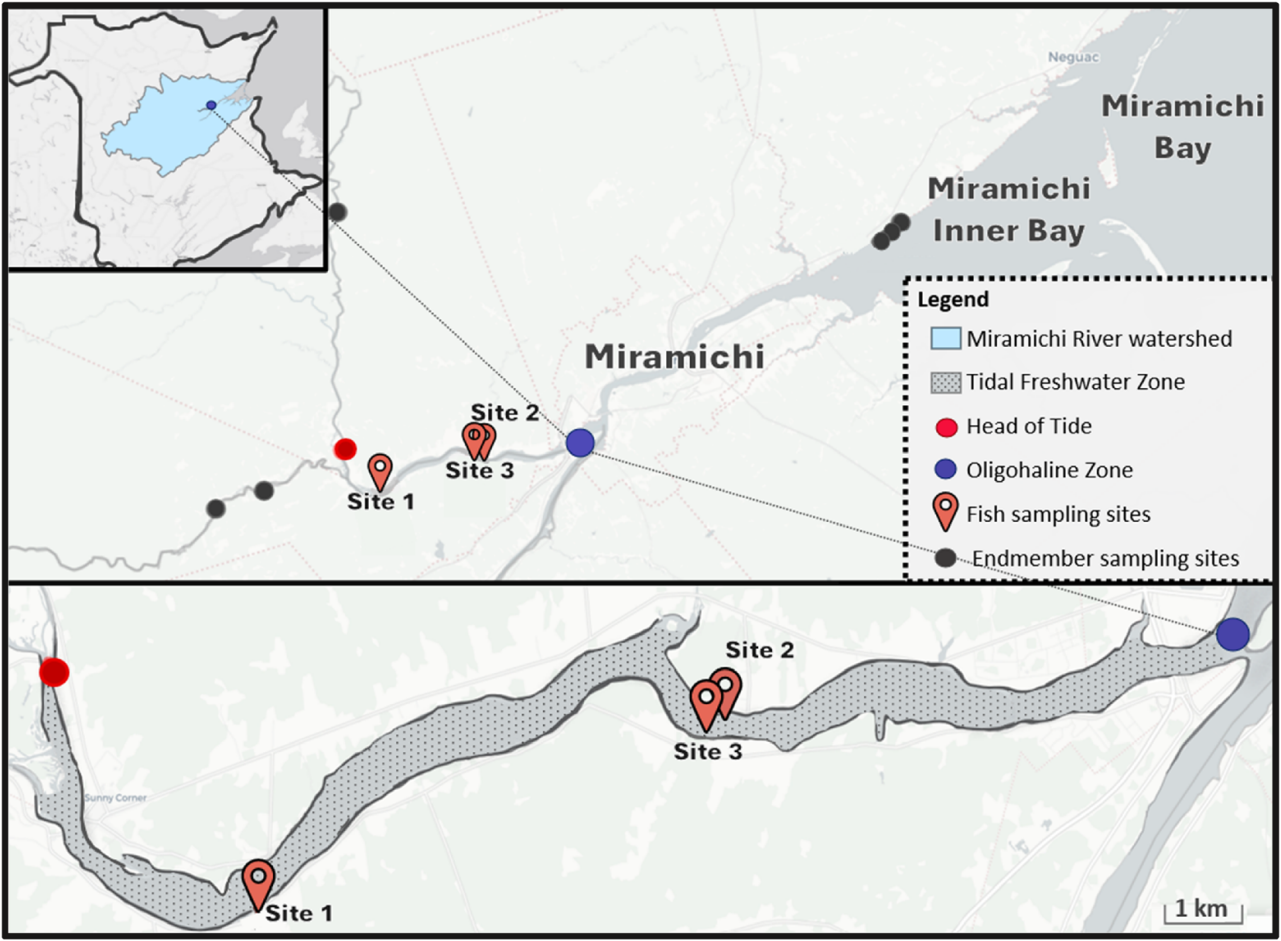
Map of the Northwest Miramichi River (New Brunswick, Canada), with the surrounding watershed indicated in the inset. The tidal freshwater zone is marked by a stippled region and situated between the head of tide (red) and the oligohaline zone (blue). Fish sampling sites are identified as Cassilis (1), Opposite Hatchett (2), and Bass Trap (3). Invertebrate endmember sampling locations are indicated in black. The inset map of New Brunswick, highlighting the Miramichi River Watershed, was generated in ArcGIS using layers provided by the Canadian Rivers Institute. All other maps were generated using OpenStreetMap data.

The Northwest Miramichi River (New Brunswick, Canada) is a main branch within the Miramichi River system that facilitates the passage of dozens of economically significant migratory and resident fishes (Bousfield, 1955; Chaput, 1995; Cunjak & Newbury, 2005). Our study took place in its tidal freshwater zone, which is fed by a network of tributaries and flows into the largest estuary in the Southern Gulf of Saint Lawrence, the Miramichi Bay (Figure 2).

To define the Northwest Miramichi's tidal freshwater zone, we marked the head of tide and the oligohaline zone (i.e., the zone where salinity increases from <0.5 ppt to between 0.5 and 5.0 ppt) using concepts laid out by Odum (1988) and local salinity and tidal data from Locke and Courtenay (1996) and Vilks and Krauel (1982). Though the location of the oligohaline zone may fluctuate due to seasonal variations in surface and bottom water salinity caused by drought, rain events, and ice melt, salinity data revealed that the area we selected as the oligohaline boundary consistently measured at expected salinity levels.

### 2.3 Field sampling

We collected fish in spring (mid‐May to early June in 2019 and 2021) during peak fish spawning and migration activity. We sampled from three sites Cassilis (1; 46.9339, −65.7836), Opposite Hatchett (2; 46.9582, −65.6899), and Bass Trap (3; 46.9583, −65.6899) to maximize our fish capture yields. Sites 1 and 3 had minimal submerged vegetation in the water and a combination of rock and sand sediments, whereas Site 2 was more sheltered with increased presence of submerged vegetation and sandier substrates.

We used multiple net types with varying mesh‐sizes across these sites to ensure we could capture a variety of fish species (mesh‐sizes of nets used at Site 1: large fyke [box, lead line, and cod = 5 mm tight, 10 mm loose]; Site 2: 6 mm beach seine; Site 3: two small fyke nets [box, lead line, and cod = 17 mm, 17 mm, and 10 mm tight, and 32 mm, 32 mm, 20 mm loose, respectively], one trap net [box = 47.6 mm and lead line = 146 mm] were used). Species captured included alewife *Alosa pseudoharengus* (Wilson 1811), blueback herring *Alosa aestivalis* (Mitchill 1814), three‐spined stickleback *Gasterosteus aculeatus* L. 1758, striped bass *Morone saxatilis* (Walbaum 1792), rainbow smelt *Osmerus mordax* (Mitchill 1814), fourspine stickleback *Apeltes quadracus* (Mitchill 1815), American eel *Anguilla rostrata* (Lesueur 1817), mummichog *Fundulus heteroclitus* (Linnaeus 1766), banded killifish *Fundulus diaphanous* (Lesueur 1817), lake chub *Couesius plumbeus* (Agassiz 1850), yellow perch *Perca flavescens* (Mitchill 1814), blacknose dace *Rhinichthys atratulus* (Herman 1804), white sucker *Catostomus commersonii* (Lacepède 1803), fall fish *Semotilus corporalis* (Mitchill 1817), common shiner *Luxilus cornutus* (Mitchill 1817), and salmon *Salmo salar* L. (smolt and parr) for a total of 16 species.

To establish a baseline isotope gradient of the Northwest Miramichi River we sampled benthic invertebrates from seven locations along the river spanning freshwater, tidal freshwater, estuarine, and marine habitats (Table S1). To obtain a representative sample of benthic invertebrates from each site, we kick‐netted 1–4 m from the shore of each location in 1‐min intervals with 5–10 intervals per site (CAB Network, 2012). In the freshwater sites, we collected stonefly nymphs (Plecoptera Perlidae), mayfly nymphs (Ephemeroptera Heptagenidae), dragonfly nymphs (Odonata, Anisoptera), and caddisfly nymphs (Trichoptera Richofilidae). In the marine sites, we collected sand shrimp (*Crangon* sp.), isopods (*Gammarus* sp.), and snails (*Littorina littorea*), the latter of which were collected haphazardly from rocks along shore (Table S4).

### 2.4 Sample storage and dissection

We humanely euthanized captured fish by immersing them in a 0.1% clove oil solution (Davis et al., 2015; Holloway et al., 2004). We identified fish species per Gautreau and Curry (2020) and sorted invertebrates based on location and taxonomic family per Merritt et al. 2008). Invertebrate and fish samples were stored on ice and then moved to a −25°C freezer within 12 h. We left most invertebrates whole (only shelling mollusks) and dissected a dorsal muscle plug from each fish (1 cm^3^) in July–September of their respective sampling year.

### 2.5 Stable isotope analyses

We oven‐dried all samples at 60°C for 48‐h then ground and weighed the resulting powder to 1 ± 0.1 mg in 5 × 3.5 mm tin capsules for analysis at the Stable Isotopes in Nature Laboratory (University of New Brunswick, Canada). Samples were flash combusted (*T* = 900°C) in an ECS 4010 elemental analyser (Costech Analytical Technologies, Valencia, CA, USA). Sulfur dioxide (SO_2_), carbon dioxide (CO_2_), and nitrogen (N_2_) were separated in a gas chromatography column (*T* = 50°C) then passed through a Delta Plus XP continuous‐flow isotope ratio mass spectrometer (Thermo Fisher Scientific, Bremen, Germany). Stable isotope ratios were measured relative to international reference materials (Vienna Pee Dee Belemnite for δ^13^C, atmospheric air for δ^15^N, and Vienna‐Canon Diablo Trilobite for δ^34^S). Instruments were calibrated to international standards using the International Atomic Energy Authority (Vienna, Austria) certified materials. Analytical error from replicating analyses (samples and reference materials) was <0.1‰. All values were normalized using secondary standards (full list in Table S2). Isotope ratios are expressed as parts per thousand (‰) and were calculated using the δ‐equation (Peterson & Fry, 1987).

### 2.6 Data analysis

We applied an arithmetic lipid‐correction to δ^13^C data (Figure S1) developed by McConnaughey and McRoy (1979) and adapted by Logan et al. (2008). We ran a fixed‐effect permutational multivariate analysis of variance (PERMANOVA) to measure the effect of year and species on our data (Oksanen, 2015; Veganv.2.6–4). We found sampling year affected consumer stable isotope ratios (*p* < 0.05), but only explained about 1% of the total variation (*F* = 20.29, *R* ^2^ = 0.01). Therefore, we chose to pool data from both years (Table S3).

### 2.7 Defining the endmembers

SIMMs infer resource use by relating the stable isotope ratios of consumers to baseline endmembers that are defined a priori using a pooled representative sample of isotopically similar invertebrates available in each habitat (Parnell et al., 2013; Post, 2002a; Post, 2002b). We defined the marine endmember using sand shrimp (*Crangon* sp., −16.90 ± 1.43, 10.58 ± 0.48, 15.19 ± 1.39, for δ^13^C, δ^15^N, and δ^34^S, respectively), isopods (*Gammarus* sp., −16.30 ± 3.24, 6.58 ± 1.56, 18.56 ± 1.44), and snails (*Littorina littorea*, −16.05 ± 0.43, 7.24 ± 0.36, 21.20 ± 0.16), and the freshwater endmember using stonefly nymphs (Plecoptera Perlidae, −24.19 ± 0.54, 4.66 ± 0.46, 9.62 ± 2.95 for δ^13^C, δ^15^N, and δ^34^S, respectively), mayfly nymphs (Ephemeroptera Heptagenidae, −22.57 ± 2.59, 3.18 ± 0.84, 10.19 ± 3.0), dragonfly nymphs (Odonata, Anisoptera, −25.13 ± 0.75, 4.25 ± 0.53, 10.40 ± 2.92), and caddisfly nymphs (Trichoptera Richofilidae, −23.02 ± 0.19, 3.83 ± 1.60, 8.63 ± 2.95). δ^13^C, δ^15^N, and δ^34^S values of the pooled freshwater and marine endmembers are −24.16 ± 1.45, 4.14 ± 0.84, and 9.92 ± 2.75 and −16.53 ± 1.94, 8.64 ± 2.36, and 17.50 ± 2.66, respectively (Table S4).

### 2.8 Trophic structure

We estimated median marine resource use with Bayesian 95% CIs using the SIMMr package, which was run for 10,000 iterations (simmr version 0.4.5; Parnell & Govan, 2019). SIMMr uses a probability‐based framework to estimate endmember contributions to the diet of each species (Govan et al., 2023). These resource use values are expressed as a proportion, where species with values closer to 1.0 can be seen as assimilating predominantly marine resources and species closer to 0.0 are more reliant on freshwater. To account for potential biases in resource use due to differences in trophic fractionation between δ^13^C, δ^34^S, and δ^15^N, SIMMr uses trophic enrichment factors (changes in δ‐ratios from prey to consumer, termed TEFs hereafter). Concentration dependence means (proportion of each element in the baseline) and TEFs taken from existing literature were used to account for model sensitivity (Table S4; Caut et al., 2009; Boecklen et al., 2011; McCutchan Jr et al., 2003; Phillips et al., 2014).

We quantified isotopic niche width using NicheRover (version 1.1.0), a Bayesian inference framework by Swanson et al. (2015) that estimates the size of species' niche region (N_R_). N_R_ is a unitless measurement representing the 95% credibility region in multivariate iso‐space, visualized as a bivariate two‐dimensional elliptical projection cast by a 3‐dimensional ellipsoid called the niche size (N_S_). N_R_ represents the posterior distribution of N_S_ for each species, calculated from 1000 Monte Carlo draw parameter list, and N_S_ is the hypervolume of N_R_.

We estimated trophic position relative to consumer prey sources using tRophicPosition (version 0.8.0) by Quezada‐Romegialli et al. (2018), a Bayesian analogue to methods by Post (2002a, 2002b) and Vander‐Zanden and Rasmussen (2001), which estimates trophic position as a random parameter with uniform prior distribution. The Bayesian dual baseline approach used in this study provides a robust estimate of consumer trophic position by incorporating δ^13^C and δ^15^N ratios from not only the consumers but also the freshwater and marine baselines (i.e., endmembers) as well as the TEFs for δ^13^C and δ^15^N, and the trophic position of baseline of each ecosystem (see Quezada‐Romegialli et al., 2018 for full breakdown of equations). Including the δ^13^C and δ^15^N ratios, TEFs, and trophic position of the freshwater and marine baselines allows the model to distinguish between different sources of nitrogen, accounting for differences in δ^13^C and δ^15^N enrichment and heterogeneity across habitats, thus correcting for potential biases in trophic position estimates caused by differing levels of δ^15^N in organisms at the base of the food web in different source habitats (Quezada‐Romegialli et al., 2018). δ^13^C and δ^15^N values of the consumers, baselines, and TEFs are modeled as random variables, with prior normal distribution on their means and uniform prior distribution on their SDs (Quezada‐Romegialli et al., 2018). We used default trophic TEFs ± SD (3.4 ± 0.98 and 0.39 ± 1.3 for nitrogen and carbon) from Post (2002a) and ran Markov chains Monte Carlo for 20,000 iterations with a burn‐in set to 2000. All three packages utilize δ^13^C and δ^15^N in their analyses, whereas resource use and isotopic niche width also incorporate δ^34^S values.

We tested for relationships between the trophic metrics using a combination of linear and quadratic regressions, per predictions made a priori. To test the influence of resource use on isotopic niche width (prediction 1) and of niche width on trophic position (prediction 2) we used quadratic regressions as we expected curvature in the relationship. To test the influence of resource use on trophic position (prediction 3), we used a linear model as we expected a linear relationship. To check if the assumptions of all three models were met, we visually inspected diagnostics plots and observed no obvious patterns in these plots (Figure S2). We then ran a Breusch Pagan test, finding no evidence of heteroscedacity (*p* > 0.05), and estimated Cook's distance, finding that some species appear to influence model fit (i.e., salmon smolt Cook's distance = 0.9 in the first model, and American eel = 1.8 in the second model), which we explore in the discussion.

To further understand how trophic position, niche area, and resource use captured similarities in species' trophic ecology, we ran a hierarchical clustering analysis using Ward's method and calculated pair‐wise Euclidean distances between the populations. We used the elbow method (i.e., plotting the within‐cluster sum of squares [WSS] for a range of cluster numbers) to determine an appropriate number of clusters. The degree of separation and overlap among clusters was identified using the Silhouette score, used here as an average for each cluster where a value close to 1 is interpreted as well‐separated, 0 suggests overlap with another cluster, and −1 indicates high overlapping across clusters, with individuals in this cluster being closer to other clusters than their own. All data were standardized prior to analyses to ensure that all variables contributed equally to the clustering.

## 3 RESULTS

### 3.1 Interspecific comparison of trophic structure

#### 3.1.1 Marine resource use

**FIGURE 3:**
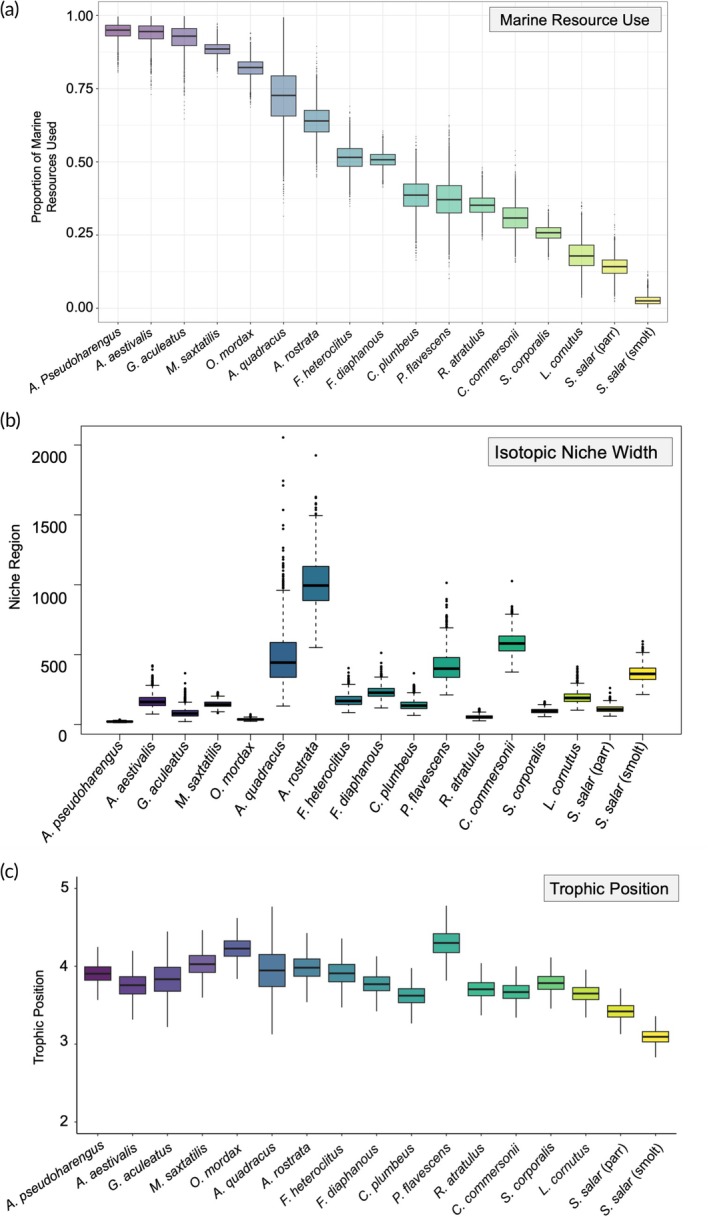
Resource use (a) isotopic niche width (b) and trophic position (c) of the 17 fish species found in the tidal freshwater zone of the Northwest Miramichi River (New Brunswick, Canada.). All trophic traits were calculated using δ^13^C and δ^15^N; resource use and niche width included δ^34^S. Fish species (*x*‐axis) are ordered from highest to lowest marine resource use.

The tidal freshwater zone community was characterized by a wide range of marine resource use estimates throughout the transition zone (Figure 3a). Atlantic salmon (Parr, Smolt), fallfish, and common shiner had low marine resource use values (*α* between 0.02 and 0.26), indicating they are more reliant on freshwater resources. Alewife, blueback herring, three‐spined stickleback, striped bass, and rainbow smelt had high marine resource use (*α* > 0.80), with most species falling in between (Figure 3a; Table 1). Unlike Atlantic salmon and fallfish, some of the freshwater species (i.e., lake chub, yellow perch, blacknose dace, and white sucker) appear to have assimilated some marine resources (Table 1). Mummichog and banded killifish had identical resource use values and assimilated an equal mix of both resources, as did fourspine stickleback and American eel, but with the two latter species showing a higher assimilation of marine resources by comparison (Figure 3a).

**TABLE 1:** Average morphometric and trophic stable isotope data across 17 fish populations.

Species	*n*	Fork length ± SD	δ^13^C ± SD	δ^15^N ± SD	δ^34^S ± SD	*α* (mean ± SD)	*α* (2.5–97.5 CI)	Niche region (±SE)	TP (mean ± SD)
*Alosa pseudoharengus* (alewife)	43	24.3 ± 1.4	−19.9 ± 0.65	12.6 ± 0.3	19.6 ± 1.5	0.94 ± 0.03	0.1	20.4 ± 3.9	3.9 ± 0.1
*Alosa aestivalis* (blueback herring)	19	22.8 ± 3.9	−20.0 ± 1.8	12.1 ± 1.1	19.3 ± 2.3	0.94 ± 0.04	0.1	167.7 ± 47.5	3.8 ± 0.2
*Gasterosteus aculeatus* (three‐spined stickleback)	8	4.8 ±0.4	−19.7 ±2.7	−12.5 ± 0.7	19.2 ± 1.7	0.92 ±0.05	0.2	85.3 ± 36.7	3.8 ± 0.2
*Morone saxatilis* (striped bass)	64	45.8 ± 11.3	−17.8 ± 1.5	14.0 ± 0.5	15.5 ± 2.3	0.89 ± 0.02	0.1	147.1 ± 21.8	4.0 ± 0.2
*Osmerus mordax* (rainbow smelt)	59	13.8 ± 1.8	−20.0 ± 0.60	13.6 ± 0.6	18.5 ± 1.4	0.82 ± 0.03	0.2	37.0 ± 6.1	4.2 ± 0.1
*Apeltes quadracus* (fourspine stickleback)	8	3.9 ± 0.6	−22.4 ± 2.7	11.7 ± 1.9	16.1 ± 2.5	0.72 ± 0.10	0.4	489.1 ± 228.4	3.9 ± 0.3
*Anguilla rostrata* (American eel)	43	54.1 ± 14.9	−21.8 ± 3.1	12.0 ± 1.7	13.2 ± 3.5	0.64 ± 0.06	0.2	1015.3 ± 193.4	4.0 ± 0.2
*Fundulus heteroclitus* (mummichog)	22	7.0 ± 2.0	−21.9 ± 2.6	11.7 ± 1.1	13.8 ± 1.2	0.51 ± 0.04	0.2	173.9 ± 47.9	3.9 ± 0.2
*Fundulus diaphanous* (banded killifish)	43	6.1 ± 0.55	−21.8 ± 1.5	11.3 ± 1.0	13.3 ± 1.9	0.51 ± 0.03	0.1	236.7 ± 45.4	3.8 ± 0.1
*Couesius plumbeus* (lake chub)	22	5.2 ± 1.7	−24.6 ± 2.3	9.9 ± 1.1	12.7 ± 1.2	0.39 ± 0.06	0.2	140.2 ± 36.4	3.6 ± 0.1
*Perca flavescens* (yellow perch)	23	19.2 ± 8.0	−23.3 ± 4.0	12.5 ± 1.4	12.6 ± 2.8	0.37 ± 0.07	0.3	413.4 ± 108.2	4.3 ± 0.2
*Rhinichthys atratulus* (blacknose dace)	24	7.8 ± 0.6	−22.5 ± 1.4	10.8 ± 0.5	12.5 ± 1.4	0.35 ± 0.04	0.1	53.6 ± 13.4	3.7 ± 0.1
*Catostomus commersonii* (white sucker)	68	18.3 ± 8.4	−24.1 ± 2.8	10.1 ± 1.5	12.1 ± 3.3	0.31 ± 0.05	0.2	583.2 ± 86.8	3.7 ± 0.1
*Semotilus corporalis* (fallfish)	45	12.9 ± 3.9	−23.6 ± 1.4	10.6 ± 0.9	11.8 ± 1.8	0.26 ± 0.03	0.1	97.4 ± 17.8	3.8 ± 0.1
*Luxilus cornutus* (common shiner)	32	5.8 ± 2.0	−24.4 ± 1.4	10.0 ± 1.0	11.9 ± 2.0	0.18 ± 0.05	0.2	193.2 ± 40.6	3.7 ± 0.1
*Salmo salar* (Parr) (Atlantic salmon)	31	6.1 ± 0.5	−24.4 ± 1.0	9.2 ± 0.8	10.9 ± 1.9	0.14 ± 0.03	0.1	110.1 ± 23.7	3.4 ± 0.1
*Salmo salar* (smolt) (Atlantic salmon)	58	13.1 ± 1.1	−25.5 ± 2.0	8.0 ± 1.2	9.1 ± 2.5	0.02 ± 0.02	0.1	369.9 ± 60.1	3.1 ± 0.1

*Note*: Variation (SD, standard error) is reported for ratios of δ^13^C, δ^15^N, and δ^34^S, consumer length, resource use, trophic position, and isotopic niche width. Sample size represented as *n*.

#### 3.1.2 Isotope niche width

Species varied in isotopic niche width. The American eel had the largest isotopic niche (1015 ± 193.4) measuring 10× greater than most species within the study. White sucker (583.2 ± 86.8), fourspine stickleback (489.1 ± 228.4), and yellow perch (413.4 ± 108.2) had the next largest niches (Figure 3b). Alewife, rainbow smelt, blacknose dace, three‐spined stickleback, and fallfish had the smallest niches (niche region <100). Isotopic niche width differed across life stages of *S. salar*, with parr having a smaller niche width than smolts (110.1 ± 23.7 and 369.9 ± 60.1, respectively). Interestingly, niche widths were lowest for species with the highest percentage of marine resource use (i.e., alewife). However, species with intermediate marine resource use had both large and small niches (Figure 3b).

#### 3.1.3 Trophic position

Trophic position of the fish community in the tidal freshwater zone was similar between species with a mean of 3.8. Trophic position ranged from the lowest for Atlantic salmon smolt to yellow perch with the highest position (Table 1), a difference of 0.8, or a little less than 1 full trophic position. Many species had similar trophic positions; for example, 4 out of 17 species had a trophic position of 3.8.

### 3.2 Relationships between trophic characteristics

We made three predictions about the relationships between resource use, niche width, and trophic position. As predicted, trophic position increased linearly with marine resource use (prediction 3; *R* ^2^ = 0.35, *p* < 0.05; Figure 3c; Table 2). However, we did not find significant evidence of a hump‐shaped (i.e., quadradic) relationship between resource use and isotopic niche width (prediction 1; *R* ^2^ = 0.11, *p* > 0.05) or isotopic niche width and trophic position (prediction 2; *R* ^2^ = 0.06, *p* > 0.05). Though the relationships we had predicted between traits were not deemed statistically significant, we were interested to observe that for a few species, some of the widest isotopic niche measurements recorded did occur at intermediate trophic positions and resource use (Figure 3). Furthermore, some species with narrow isotopic niches occur at low and high trophic positions and marine resource use values. Unexpectedly, however, we also found some of the species with the smallest isotopic niches at intermediate resource use and trophic position, which differed from our original predictions (Figure 3).

**TABLE 2:** Series of three models measuring for (1) a quadratic association between resource use (*α*) and isotopic niche width (INW); (2) a quadratic association between isotopic niche width and trophic position (TP); and (3) a linear association between resource use and trophic position for 17 fishes.

	Estimate	Standard error	*T*‐value	*p*‐Value
1. *a* and INW				
Intercept	106.5	210.2	0.506	0.620
Resource use	996.5	933.5	1.067	0.304
Resource use^2^	−1032.7	853.9	−1.209	0.247
*R* ^2^ = 0.11				
2. INW and TP				
Intercept	3.86e+00	1.4e‐01	27.0	1.75 e‐13
Isotopic niche width	−6.1‐04	8.87e‐04	−0.688	0.503
Isotopic niche width^2^	7.61e‐07	8.95e‐07	0.850	0.410
*R* ^2^ = 0.06				
3. *a* and TP				
Intercept	3.51	0.11	30.74	5.8e‐15
Resource use	0.54	0.19	2.85	0.0122
*R* ^2^ = 0.35				

*Note:* Estimated values across predictions 1, 2, and 3 reflect differences in variable scale.

Abbreviations: INW, isotopic niche width; TP, trophic position.

**FIGURE 4:**
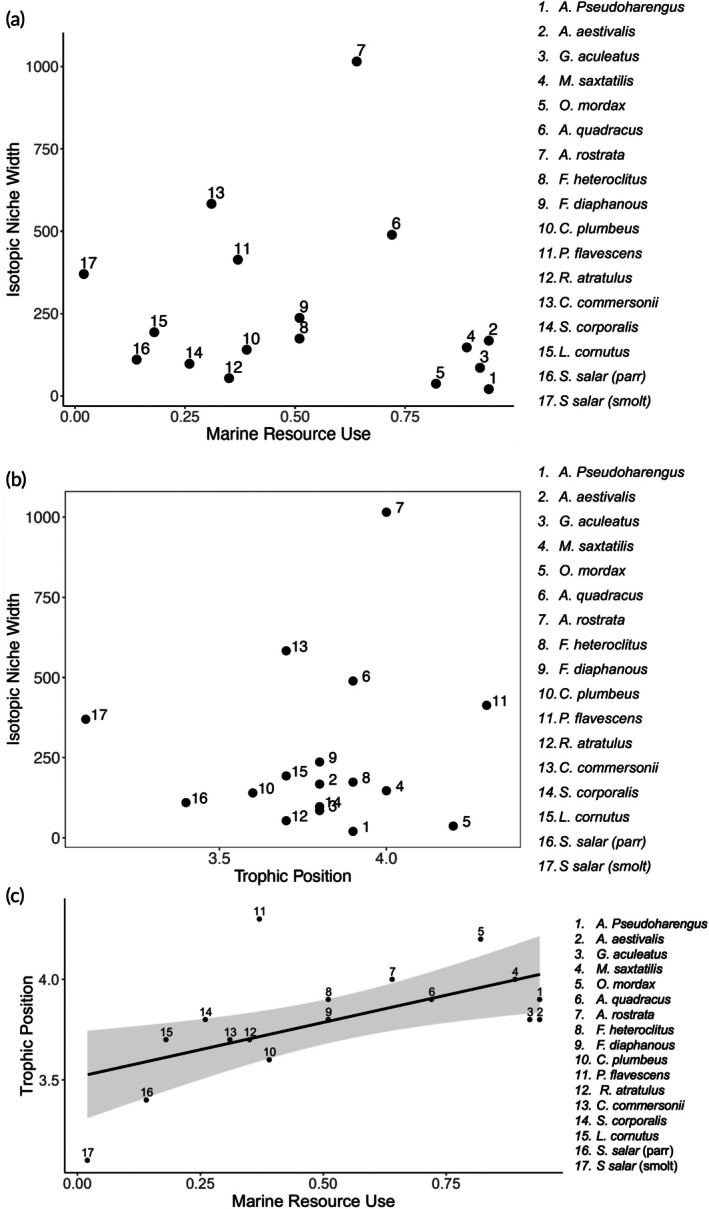
Comparison of resource use to isotopic niche width (a), isotopic niche width to trophic position (b), and resource use to trophic position (c) for 17 species of fish. Linear trendline is fitted to the linear regression comparing marine resource use to trophic position (a), and shading represents standard error.

We continued to explore these results through hierarchical clustering analysis. We found the marine migrants (e.g., alewife, blueback herring, three‐spined stickleback, striped bass, and rainbow smelt) and many freshwater species (e.g., lake chub, blacknose dace, fall fish, common shiner, and Atlantic salmon [smolt, parr]) each formed a cluster with moderate separation (Silhouette scores = 0.58 and 0.35, respectively). Clustering was less resolute in species integrating a mixed subset of resources. The American eel (intermediate resource use, trophic position, and wide isotopic niche width) was assigned to its own cluster, and the fourspine stickleback, mummichog, banded killifish, yellow perch, and white sucker formed another, but individuals in these clusters were not well separated from other clusters as determined by Silhouette scores close to 0 (Silhouette scores 0.00 and 0.13, respectively). Low cluster separation across the more intermediate consumers highlights the complexity of the fish community in the tidal freshwater habitat (Figure 4).

## 4 DISCUSSION

Freshwater and marine ecosystems are linked through the trophic interactions of fishes. Yet, there is limited information on the food web structure of tidal freshwater zones, a transitional habitat at the upstream end of a river's estuary that is situated between these two ecosystems. We addressed this knowledge gap, using stable isotopes to describe the trophic structure (e.g., resource use, isotope niche width, trophic position) of 17 fish populations occupying the tidal freshwater zone of the Northwest Miramichi River. We found a diverse fish community comprising both resident and migrant species. Migrant species predominantly relied on freshwater or marine resources (depending on whether they were migrating from or to the ocean) and had relatively narrow niches, whereas some species integrated a combination of these resources. Species' incorporating resources from both sources had a wide range in niche widths, some being narrower and others among the widest observed. Moreover, trophic position increased with an increase in percentage use of marine resources. These results provide new insights into the trophic structure of fishes in the tidal freshwater zone, suggesting these fish exhibit a wide variety of trophic ecologies which we explore below.

### 4.1 Fish community trophic structure

All fish in the study were captured within the same general area of the tidal freshwater zone. Yet, many of the diadromous species' diets were reflective of adjacent freshwater or marine habitats, suggesting diadromous species maintained a trophic structure that resembles their habitat of origin. For example, the Atlantic salmon smolts were caught during their seaward migration and had freshwater resource use, a narrow isotopic niche, and a relatively low trophic position. These results suggest that smolts had not fed much during migration, relying instead on their upstream invertebrate diet. However, it is also plausible that smolt do not remain in the tidal freshwater zone long enough for a change in the isotope ratios of their muscle tissues to occur (Jardine et al., 2005; Thorstad et al., 2012). Both possibilities would further explain observed trophic differences between the salmon smolt and parr life stages, with parr showing a slight increase in marine resource use compared to smolts, as it is likely that the parr were not fasting and are also residents of the tidal freshwater zone.

The other anadromous migrants (alewife, blueback herring, rainbow smelt, and striped bass) feed and grow in the ocean and outer estuary and use freshwater river habitat to spawn in the spring. These fishes integrated a high proportion of marine resources with similarly narrow niches but had higher trophic positions than smolts, supporting the positive relationship we observed between marine resource use and trophic position. These trophic‐level differences may be due to differences in food chain length across marine to freshwater river food webs (Post, 2002b; Sánchez‐Hernández & Amundsen, 2018; Vander Zanden & Fetzer, 2007). The marine anadromous species retaining the isotopic signature of their origin habitat suggests they may also be fasting, or that the timing of their arrival to the tidal freshwater habitat may mean their tissues do not yet reflect more recent resource use trends (Heady & Moore, 2012).

In contrast, some species had a trophic status that was more reflective of a mixture of the freshwater and marine habitats. American eel reproduce and begin their life in the marine environment and migrate to freshwater to grow and mature (i.e., catadromous). These eels undergo seasonal feeding migrations between freshwater, brackish, and estuarine regions (Chino & Arai, 2009; Clément et al., 2014; Daverat & Tomás 2006; Harrod et al., 2005; Jessop et al., 2008; Sweezey, 2014; Wright et al., 2022). We believe such behavioral differences are reflected in the trophic structure of eels captured in the tidal freshwater zone, which had intermediate resource use and the largest isotopic niche width. The notion that the American eel's trophic structure differed from that of the anadromous species described above is weakly reinforced by the results of our hierarchical clustering analyses, which separated the American eel from all other fishes but suggested there was potential for overlap with other groups.

Interestingly, after the American eel, the yellow perch (*P. flavescens*) and white sucker (*C. commersonii*) had the next largest niches, assimilated a mixture of freshwater and marine, and consequently were not grouped with the other freshwater fishes in the cluster analysis. Though white sucker are a resident benthic feeding species with high site fidelity, they undergo freshwater migrations in spring for feeding and breeding, covering distances as far as 40 km (Doherty et al., 2010; Hanson & Courtenay, 1995; Scott & Crossman, 1973). These seasonal migrations may be contributing to the white sucker's large niche width and broader assimilation of resources. The mummichog and banded killifish (*F. heteroclitus and F. diaphanous*) both had intermediate resource use but with narrow isotopic niches. These results suggest less individual variation in their trophic status.

### 4.2 Relationship between trophic characteristics

To further understand the trophic structure in the tidal freshwater zone we tested for patterns among resource use, niche width, and trophic position. We found that trophic position varied less than one trophic level among fish community members, but per previously published literature it increased with percentage marine resource use (Sánchez‐Hernández & Amundsen, 2018). There are several explanations for why this pattern between resource use and trophic position was observed. Increased size prey range and resource availability within marine environments is thought to increase food chain length in marine ecosystems, which may allow for higher‐level trophic consumers to specialize on higher‐level trophic prey compared to freshwater river ecosystems (Post, 2002b; Vander Zanden & Fetzer, 2007).

Some expected patterns were not observed, causing us to re‐examine the initial predictions (i.e., a hump‐shaped relationship between resource use and isotopic niche width, and isotopic niche width and trophic position). Conceptually, it seems reasonable that having narrow resource use (i.e., highly freshwater or marine) would constrain consumer niche width and that having a small niche width would be associated with well‐defined trophic positions (i.e., high, or low). It is equally reasonable that isotopic niche width would expand as the result of a species broadly integrating prey from different habitats. Such feeding behavior should result in a more intermediate trophic position on average. We did observe that most freshwater and marine fishes with small niches had lower and higher trophic positions and that many species had high variation in all three metrics. However, we also observed species with narrow niches at intermediate resource use and intermediate trophic positions, including *F. heteroclitus* and *F. diaphanous*. Our results suggest that within the tidal zone, banded killifish and the mummichog may be capable of acting as a sort of “specialist” population, specializing on (i.e., exclusively integrating) some intermediate resource pool found within the tidal freshwater zone. Alternatively, it is also possible that individual fish of each population are coupling marine and freshwater resources in similar ways, causing their niche width to remain small amid intermediate resource use. Without data comparing the diets of individual consumers, such questions remain unanswered. However, the notion that isotopic niche expands in response to broader resource use (and can, by extension, lead to trophic omnivory) remains true for some species. We visually observed this when comparing the relationship between these metrics. Many species (i.e., American eel, white sucker, fourspine stickleback, yellow perch) appeared to cluster around a distinct “middle” zone of intermediate resource use, trophic position, and isotopic niche width values. However, because other species (banded killifish, mummichog) do not follow this pattern due to their intermediate resource use and narrow isotopic niche width, the strength of the relationship between these metrics is decreased. We cannot explain the observed fish community trophic data with a simple hump‐shaped curvilinear model or by membership to distinct clusters in trophic space. The trophic roles of consumers in the freshwater tidal zone operate more on a continuum rather than a distinct cluster.

### 4.3 Limitations and future considerations

Stable isotopes are a valuable tool for revealing long‐term trophic information among consumers with distinct prey sources, but whether insights on dietary and ecological niches can be inferred from estimates of isotopic niches is still under scrutiny. A species' isotopic niche is influenced by variation in the isotopic baseline of its ecosystem, requiring variation to be pronounced enough to distinguish between resource pools (Hette‐Tronquart, 2019). However, when single prey species vary widely in isotope value due to location differences and when two different prey species appear isotopically similar at a particular location, relating isotopic niche to dietary niche becomes challenging. Our results show this issue may be particularly pertinent in tidal freshwater transition zones. Such concerns have been taken up by other researchers (Hette‐Tronquart, 2019; Sheppard et al., 2018). Though the goal of our study was centered on characterizing habitat use of a tidal freshwater zone during a period of high fish activity, future trophic studies in transition zones may benefit from a time‐integrated approach that considers intraspecific dietary differences among individual consumers (Araújo et al. 2011; Bolnick et al., 2003). Taking a temporal approach, incorporating size and age class data, and additional dietary analyses (i.e., DNA metabarcoding or gut content) may showcase a seasonal dynamics in trophic structure in tidal freshwater zones and resolve whether species with large isotopic niches simply target similar prey at different points of the river continuum, or whether populations truly consume a large variety in prey species.

## 5 CONCLUSIONS

In this study, we captured a snapshot of the tidal freshwater zone food web during a critical window of high community interaction between fishes. We found that fishes varied in their trophic status and that the relationship between different trophic metrics differed from predictions based on existing research. In spring, the tidal freshwater zone erupts with life as migrant species descend downstream from their respective tributaries, or swim upstream from various marine and estuarine habitats. Here, these migrants coalesce with resident species in this small stretch of the river continuum. The tidal freshwater zone not only provides a physical landscape required for habitat coupling in the form of tidal fluctuations, but it also supports such coupling by the migrant fishes that move resources, nutrients, and organic matter between the broader freshwater and marine food webs (Bauer & Hoye, 2014). This study contributes information on the trophic status of resident and migrant fish species to understand the food web connections within transition zones and across adjacent ecosystems.

## AUTHOR CONTRIBUTIONS

All authors contributed to the study design. Emma E. Bowser collected the samples with the help of Fisheries and Oceans colleagues including Cindy Breau and Tyler D. Tunney. Emma E. Bowser analysed and interpreted the data with guidance from Brian Hayden and Tyler D. Tunney. Emma E. Bowser wrote the first draft of the manuscript, and all authors contributed to revisions used to create the final version. Cindy Breau obtained sampling permits as part of a collection of research projects on the Northwest Miramichi River.

## FUNDING INFORMATION

Funding was provided by the Freshwater Habitat Initiative and Fisheries and Oceans Canada Competitive Science Research Fund granted to C. B. in addition to her research funds. E. B. was supported by an NSERC DG award (RGPIN‐2019‐04783) granted to B. H, as well as funds from Fisheries and Oceans Canada National Centre for Effectiveness Science Freshwater Habitat Initiative granted to T. D. T.

## Supporting information


**Figure S1.** Normalization model for arithmetic lipid correction on fish muscle tissue using a formula developed by McConnaughey and McRoy (1979) and adapted by Logan et al. (2008).

**Figure S2.** Diagnostic plots for a series of three regression analyses measuring for (1) a linear association between resource use (*α*) and isotopic niche width (INW); (2) a quadratic association between isotopic niche width and trophic position (TP); and (3) a quadratic association between resource use and trophic position for 17 fishes. Within each plot, four diagnostic panels are displayed, showcasing (1) residuals versus fitted values, (2) normal Q‐Q plot of residuals, (3) scale‐location plot, and (4) residuals versus leverage. These diagnostic plots provide insights into the assumptions and goodness‐of‐fit of each regression model prior to analysis.

**Figure S3.** Hierarchical clustering dendrogram showing the relationships among the trophic characteristics (resource use, isotopic niche width, trophic position) of 17 fish populations in the Northwest Miramichi River (New Brunswick, Canada). The *Y*‐axis represents the linkage distance, and the branches indicate cluster formation at different thresholds. Clusters were generated using Ward's method, and pair‐wise Euclidean distances were calculated between the populations. Silhouette values for clusters 1–4 are 0.58, 0.13, 0.00, and 0.35, respectively.

**Table S1.** Invertebrate and fish sampling locations from the 2019 and 2021 sampling seasons. All coordinates were taken from sampling locations along the Miramichi River tidal gradient.

**Table S2.** List of standards used in stable isotopes or carbon, nitrogen, and sulfur analyses comparing expected (exp) and measured (meas) values.

**Table S3.** A test of species and year as predictors of variation in stable isotope ratios using fixed effect permutational multivariate analysis of variance (PERMANOVA) based on the McArdle and Anderson method of sum of squares.

**Table S4.** Stable isotope values of the invertebrate groups are used to generate the freshwater and marine endmembers for the stable isotope mixing model. Freshwater and marine endmember values were generated using the mean and SD of δ^13^C, δ^15^N, and δ^34^S values of a representative sample of near‐shore benthic invertebrates sourced from freshwater and marine locations. In order to maximize available biological material for stable isotope analyses, individual specimens were pooled into test tubes, with each sampling tube containing 5–10 individuals of a given taxon. Therefore, # of vials refers to the number of tubes submitted per taxa and not the total number of specimens.

**Table S5.** Concentration dependence (proportion of each element in the baseline) and trophic enrichment factor (changes in δ‐ratios from prey to consumer, abbreviated to TEF) values sourced from McCutchan Jr et al. (2003) implemented into stable isotope mixing model resource use analyses.
