# Supplementary figures and images for: Resource use, niche width, and trophic position reveal diverse trophic structure in a tidal freshwater zone fish community
Source: J Fish Biol. 2025 Feb 25;106(6):1876–88. doi: 10.1111/jfb.16057 (PMC12244314; doi:10.1111/jfb.16057)

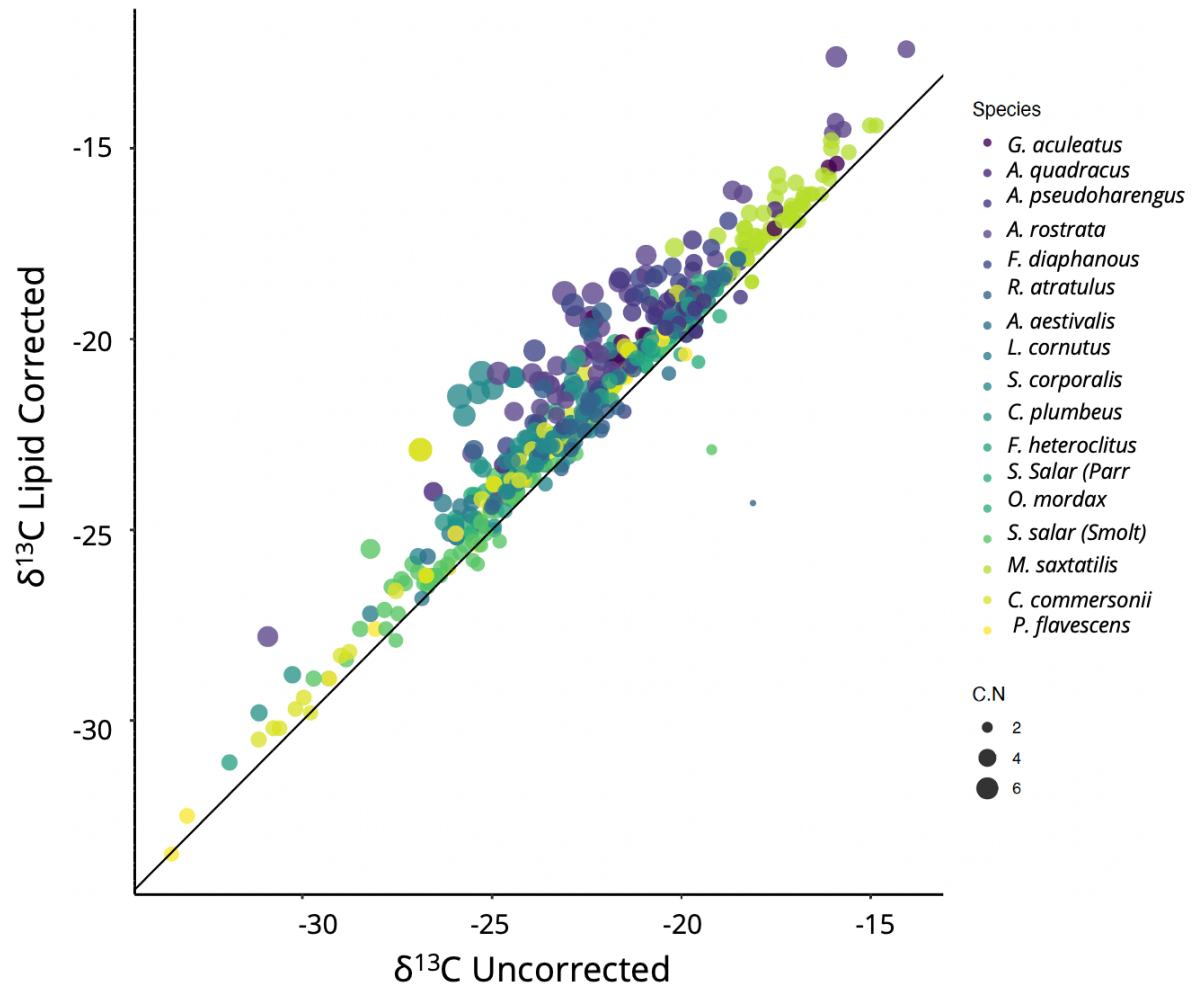

Supplement: Supplementary file 1 — Figure S1. Normalization model for arithmetic lipid correction on fish muscle tissue using a formula developed by McConnaughey and McRoy (1979) and adapted by Logan et al. (2008). [file JFB-106-1876-s003.pdf]

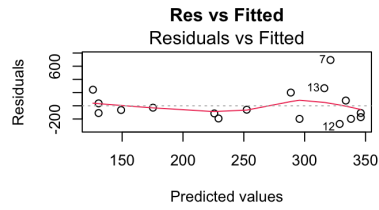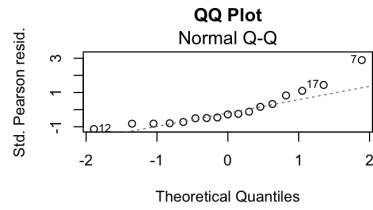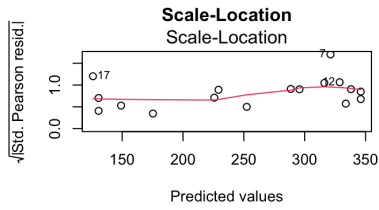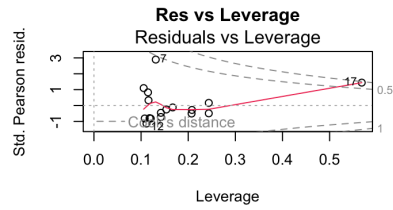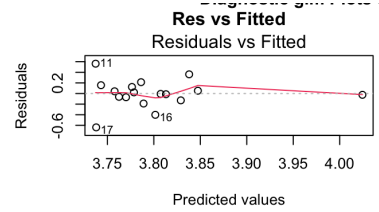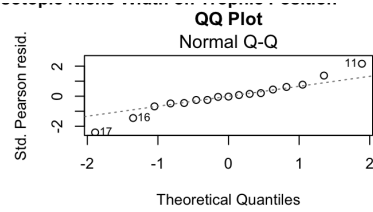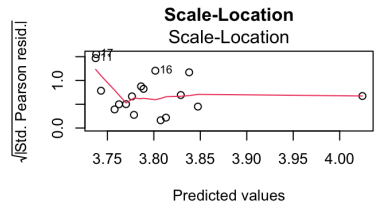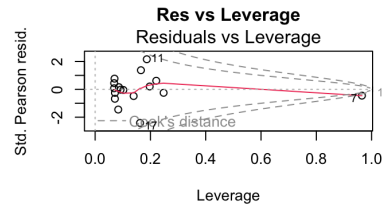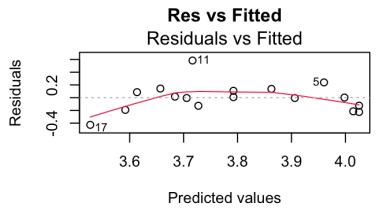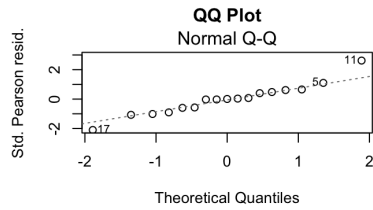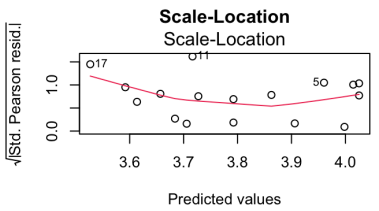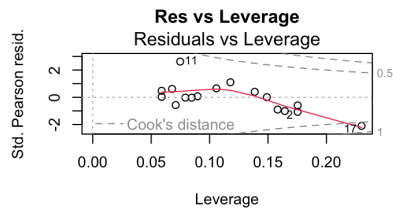

Supplement: Supplementary file 2 — Figure S2. Diagnostic plots for a series of three regression analyses measuring for (1) a linear association between resource use (α) and isotopic niche width (INW); (2) a quadratic association between isotopic niche width and trophic position (TP); and (3) a quadratic association between resource use and trophic position for 17 fishes. Within each plot, four diagnostic panels are displayed, showcasing (1) residuals versus fitted values, (2) normal Q‐Q plot of residuals, (3) scale‐location plot, and (4) residuals versus leverage. These diagnostic plots provide insights into the assumptions and goodness‐of‐fit of each regression model prior to analysis. [file JFB-106-1876-s005.pdf]

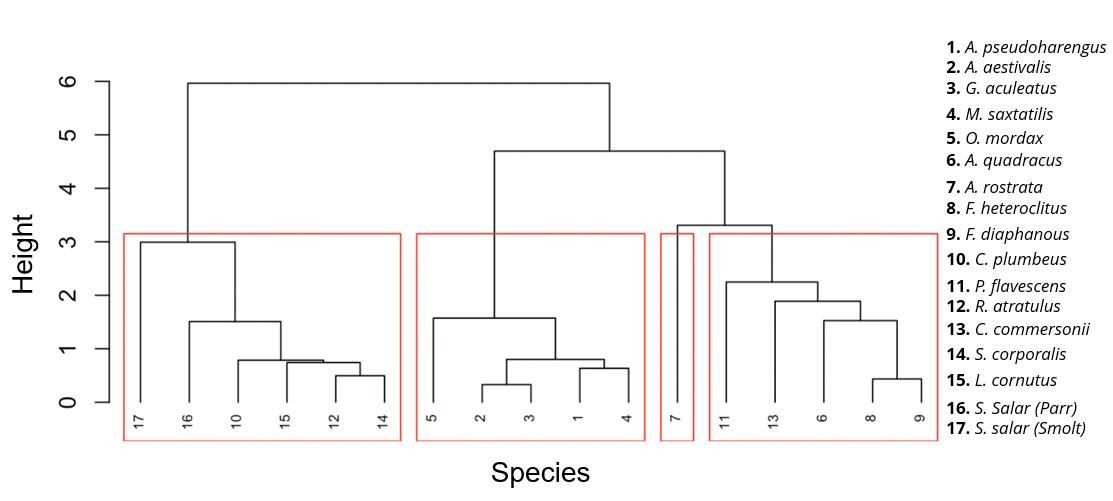

Supplement: Supplementary file 3 — Figure S3. Hierarchical clustering dendrogram showing the relationships among the trophic characteristics (resource use, isotopic niche width, trophic position) of 17 fish populations in the Northwest Miramichi River (New Brunswick, Canada). The Y‐axis represents the linkage distance, and the branches indicate cluster formation at different thresholds. Clusters were generated using Ward's method, and pair‐wise Euclidean distances were calculated between the populations. Silhouette values for clusters 1–4 are 0.58, 0.13, 0.00, and 0.35, respectively. [file JFB-106-1876-s001.docx]
