# Supplementary material for: Resource use, niche width, and trophic position reveal diverse trophic structure in a tidal freshwater zone fish community
Source: J Fish Biol. 2025 Feb 25;106(6):1876–88. doi: 10.1111/jfb.16057 (PMC12244314; doi:10.1111/jfb.16057)
Supplement: Supplementary file 4 — Table S1. Invertebrate and fish sampling locations from the 2019 and 2021 sampling seasons. All coordinates were taken from sampling locations along the Miramichi River tidal gradient. [file JFB-106-1876-s002.docx]

| **Location** | **Sample Collected** | **Habitat** | **Year** | **Latitude** | **Longitude** |
| --- | --- | --- | --- | --- | --- |
| Little South-West | Invertebrate | Freshwater | 2019 | 46.9234 | -65.9583 |
| Lyttleton | Invertebrate | Freshwater | 2021 | 46.9358 | -65.9085 |
| Wayerton Bridge | Invertebrate | Freshwater | 2019 | 47.1354 | -65.8332 |
| Wilson’s Point | Invertebrate | Estuary | 2019 | 46.9640 | -65.58185 |
| Cassilis | Fish | Freshwater  Tidal Zone | 2019 2021 | 46.9339 | -65.7836 |
| Bass Trap | Fish | Freshwater  Tidal Zone | 2019 2021 | 46.9583 | -65.6899 |
| Opposite Hatchett | Fish | Freshwater Tidal Zone | 2019 2021 | 46.9582 | -65.6899 |
| Strawberry Marsh | Invertebrate | Estuary | 2019 2021 | 46.9881 | -65.5631 |
| Beaubear Island | Invertebrate | Estuary | 2021 | 46.9785 | -65.5553 |
| Bartibog | Invertebrate | Estuary | 2021 | 47.0746 | -65.3363 |
| East Point | Invertebrate | Marine | 2021 | 47.0744 | -65.3353 |
| Oak Point | Invertebrate | Marine | 2019 2021 | 47.1280 | -65.2553 |
| Pointe-Aux-Carr | Invertebrate | Marine | 2021 | 47.0771 | -65.2152 |
