# Supplementary material for: Resource use, niche width, and trophic position reveal diverse trophic structure in a tidal freshwater zone fish community
Source: J Fish Biol. 2025 Feb 25;106(6):1876–88. doi: 10.1111/jfb.16057 (PMC12244314; doi:10.1111/jfb.16057)
Supplement: Supplementary file 5 — Table S2. List of standards used in stable isotopes or carbon, nitrogen, and sulfur analyses comparing expected (exp) and measured (meas) values. [file JFB-106-1876-s004.docx]

|  | Exp.  dC | Meas.  dC | Exp.  dN | Meas.  dN | Exp.  dS | Meas.  dS | N |
| --- | --- | --- | --- | --- | --- | --- | --- |
| Nico-tanimide | -32.5 | -32.5 ± 0.1 | 2 | 2.0 ± 0.04 |  |  | 12 |
| Sulfan-ilimide | -25.8 | -25.7 ± 0.2 | -1.5 | -1.4 ± 0.1 | 15.3 | 15.2 ± 0.4 | 61 |
| Meth-ionine | -29.4 | -29.2 ± 0.6 | 0.15 | 0.2 ± 0.2 | 8.3 | 8.1 ±  0.6 | 23 |
| SSS | -16.7 | -16.7 ± 0.4 | 12.4 | 12.4 ± 0.06 | 20.5 | 21.8 ± 0.4 | 46 |
| BCS | -27.4 | -27.4 ± 0.08 | 11 | 11.0 ± 0.05 | 5.9 | 6.3 ±  0.5 | 47 |
| BLS | -18.8 | -18.7 ± 0.08 | 7.17 | 7.1 ± 0.09 | 7.4 | 8.0 ±  0.7 | 55 |
| MLS | -22.3 | -22.3 ± 0.07 | 14 | 14.0 ± 0.07 | 13.7 | 14.7 ± 0.4 | 68 |
| USGS61 | -35.05 | -35.1 ± 0.13 | -2.87 | -2.8 ± 0.04 |  |  | 55 |
| S1 |  |  |  |  | 0.3 | -0.2 ± 0.4 | 48 |
| S2 |  |  |  |  | 22.6 | 22.7 ± 0.3 | 48 |
| CH7 | -32.15 | -32.1 ± 0.01 |  |  |  |  | 3 |
| N2 |  |  | 20.3 | 20.4 ± 0.17 |  |  | 3 |
