# Supplementary material for: Resource use, niche width, and trophic position reveal diverse trophic structure in a tidal freshwater zone fish community
Source: J Fish Biol. 2025 Feb 25;106(6):1876–88. doi: 10.1111/jfb.16057 (PMC12244314; doi:10.1111/jfb.16057)
Supplement: Supplementary file 6 — Table S3. A test of species and year as predictors of variation in stable isotope ratios using fixed effect permutational multivariate analysis of variance (PERMANOVA) based on the McArdle and Anderson method of sum of squares. [file JFB-106-1876-s007.docx]

|  | **Degrees of Freedom** | **Sum of Squares** | **R^2^** | **F-Statistic** | **P value** |
| --- | --- | --- | --- | --- | --- |
| **Species** | 16 | 3.34 | 0.67 | 80.79 | 0.001 *** |
| **Year** | 1 | 0.05 | 0.01 | 20.29 | 0.002 ** |
| **Species:Year** | 11 | 0.10 | 0.02 | 3.60 | 0.001 *** |
| **Residual** | 583 | 1.51 | 0.30 |  |  |
