# Supplementary material for: Resource use, niche width, and trophic position reveal diverse trophic structure in a tidal freshwater zone fish community
Source: J Fish Biol. 2025 Feb 25;106(6):1876–88. doi: 10.1111/jfb.16057 (PMC12244314; doi:10.1111/jfb.16057)
Supplement: Supplementary file 7 — Table S4. Stable isotope values of the invertebrate groups are used to generate the freshwater and marine endmembers for the stable isotope mixing model. Freshwater and marine endmember values were generated using the mean and SD of δ13C, δ15N, and δ34S values of a representative sample of near‐shore benthic invertebrates sourced from freshwater and marine locations. In order to maximize available biological material for stable isotope analyses, individual specimens were pooled into test tubes, with each sampling tube containing 5–10 individuals of a given taxon. Therefore, # of vials refers to the number of tubes submitted per taxa and not the total number of specimens. [file JFB-106-1876-s006.docx]

| **Habitat** | **Invertebrate Group** | **# of Vials** | **δ^13^C ± SD** | **δ^15^N ± SD** | **δ^34^S ± SD** |
| --- | --- | --- | --- | --- | --- |
| Freshwater | **Stonefly nymphs**  (Plecoptera Perlidae) | 7 | -24.19±  0.54 | 4.66±  0.46 | 9.62±  2.95 |
| Freshwater | **Mayfly nymphs**  (Ephemeroptera Heptagenidae) | 4 | -22.57±  2.59 | 3.18±  0.84 | 10.19±  3.0 |
| Freshwater | **Dragonfly nymphs**  (Odonata Anisoptera) | 10 | -25.13±  0.75 | 4.25±  0.53 | 10.40±  2.92 |
| Freshwater | **Caddisfly nymphs**  (Trichoptera Richofilidae) | 3 | -23.02±  0.19 | 3.83±  1.60 | 8.63±  2.95 |
| **Freshwater** | **Endmember** |  | -24.16±  1.45 | 4.14±  0.84 | 9.92±  2.75 |
| Marine | **Shrimp**  *Crangon. sp* | 8 | -16.90±  1.43 | 10.58±  0.48 | 15.19±  1.39 |
| Marine | **Snail**  *Littorina*  *litterea* | 3 | -16.05±  0.43 | 7.24±  0.36 | 21.20±  0.16 |
| Marine | **Isopods**  (*Gammarus sp*.) | 7 | -16.30±  3.24 | 6.58±  1.56 | 18.56±  1.44 |
| **Marine** | **Endmember** |  | -16.53±  1.94 | 8.64±  2.36 | 17.50±  2.66 |
