# Supplementary material for: Resource use, niche width, and trophic position reveal diverse trophic structure in a tidal freshwater zone fish community
Source: J Fish Biol. 2025 Feb 25;106(6):1876–88. doi: 10.1111/jfb.16057 (PMC12244314; doi:10.1111/jfb.16057)
Supplement: Supplementary file 8 — Table S5. Concentration dependence (proportion of each element in the baseline) and trophic enrichment factor (changes in δ‐ratios from prey to consumer, abbreviated to TEF) values sourced from McCutchan Jr et al. (2003) implemented into stable isotope mixing model resource use analyses. [file JFB-106-1876-s008.docx]

| Habitat |  |  | δ^13^C  ± SD | δ^15^N ± SD | δ^34^S  ± SD |
| --- | --- | --- | --- | --- | --- |
| Freshwater | TEF |  | 0.4 ± 0.12 | 2.0 ± 0.2 | 0.4 ± 0.52 |
| Freshwater | Concentration Dependence |  | 0.37 ±0.06 | 0.09 ±0.003 | 0.09 ±0.03 |
| Marine | TEF |  | 0.4 ± 0.12 | 2.0 ± 0.2 | 0.4 ± 0.52 |
| Marine | Concentration Dependence |  | 0.47 ±0.02 | 0.11 ±0.01 | 0.11 ±0.01) |
